# Handheld Bioprinters in Skin Regeneration: Current Landscape, Clinical Promise, and the Road Ahead

**DOI:** 10.3390/biomedicines14092021

**Published:** 2026-09-08

**Authors:** Andrey Kolosov, Yana Khristidis, Daria Revokatova, Polina Bikmulina, Boris Ershov, Raisa Chilova, Anna Solovieva, Peter Timashev, Anastasia Shpichka

**Affiliations:** 1 Institute for Regenerative Medicine, Sechenov University, Moscow 119991, Russia; 2 Department of Obstetrics and Gynaecology No 1, Sechenov University, Moscow 119991, Russia; 3 Department of Polymers and Composites, N.N. Semenov Federal Research Center for Chemical Physics, Russian Academy of Sciences, Moscow 119991, Russia; 4 Department of Biological Chemistry, Sechenov University, Moscow 105043, Russia

**Keywords:** portable bioprinter, in situ bioprinting, skin regeneration, tissue engineering, wound healing, clinical trials

## Abstract

Portable handheld bioprinters represent a transformative advancement in personalized skin regeneration, bypassing the logistical constraints of stationary lab-based systems by enabling real-time, in situ fabrication of bioengineered constructs directly within the wound bed. This review aims to evaluate the current state of their development and clinical translation. One of the foci is placed on the stringent physicochemical requirements for bioinks, where we examined the critical balance between bioadhesion—facilitated by functional groups—and mechanical cohesion necessary for maintaining structural integrity during deposition, while RGD motifs are considered primarily as promoters of integrin-mediated cell adhesion. Preclinical studies have demonstrated promising effects of bioprinted constructs on wound healing and tissue organization; however, human evidence for handheld and direct in situ skin bioprinting remains limited, and clinical efficacy has yet to be established in controlled studies. Nevertheless, widespread adoption is hindered by inferior printing fidelity relative to stationary counterparts, a lack of standardized GMP-compliant bioink production, and regulatory ambiguity that impedes clear classification as either medical devices or biologics. Practical barriers, including intraoperative sterility assurance and operator training, also remain unresolved. Looking ahead, we discuss how the convergence of, in particular, artificial intelligence for real-time wound morphometry, closed-loop process control, and smart, self-healing biomaterials promises to surmount these obstacles. We conclude that these synergistic innovations may propel handheld bioprinters from experimental prototypes toward clinical tools with the potential to reshape reconstructive surgery and emergency wound care, although their clinical value will require validation in appropriately designed human studies.

## 1. Introduction

Among the diverse applications of 3D bioprinting, skin tissue engineering has attracted considerable interest owing to the high global prevalence of chronic wounds, burns, and injuries that result in skin loss [1,2]. Although significant advances have been made in the design and operation of stationary bioprinters—enabling the fabrication of highly defined constructs under well-controlled conditions—their clinical translation remains limited. These systems are typically bulky, non-portable, and confined to the laboratory, which enables only ex vivo fabrication of constructs, i.e., outside their intended site of application. In contrast, portable bioprinters are powered by a battery pack or incorporate other engineering solutions that make them transportable [3]. This opens opportunities for point-of-care (POC) bioprinting or point-of-care biomaterial fabrication—the direct, in situ delivery of bioink to a wound defect site [4,5,6]. Such systems may be automated, utilizing a robotic arm fitted with a bioprinter head (robotic in situ bioprinting) [7,8], or handheld and manually operated, with the operator directly positioning the printing device [2,7].

Handheld portable bioprinters are emerging as a rapidly evolving alternative that overcomes limitations of stationary bioprinters and some large robotic in situ bioprinters. These devices enable real-time, in situ deposition of bioengineered constructs directly onto the wound surface, allowing for immediate coverage and promoting tissue regeneration at the injury site. Several early-stage prototypes have already demonstrated promising results in preclinical models [1,9,10]. Moreover, hybrid handheld platforms that integrate extrusion, spray-based cell deposition (cell delivery through spray instruments), and electrospinning further enhance functional versatility [11]. These advances position handheld bioprinters as potentially powerful medical tools in emergency medicine, burn care, and battlefield or remote-area settings, where conventional wound management approaches may fall short [9,12].

However, several challenges still hinder the clinical adoption of handheld bioprinters. First, their printing resolution and structural accuracy remain inferior to those of stationary platforms, raising concerns about the fidelity and stability of printed tissues [13]. Second, the biocompatibility, mechanical stability, and standardization of bioinks tailored for portable devices are still under development, and there is a lack of consensus on optimal formulations for skin regeneration [14]. Third, regulatory uncertainties continue to impede market access: handheld bioprinters do not clearly fit into established medical device classifications, and clinical validation pathways are still underdeveloped [14,15]. Moreover, operational barriers—including the need for sterile printing conditions, limited operator training, and the challenge of integration into clinical workflows—further complicate practical deployment [14].

Previous reviews have established the technological foundations of handheld and in situ bioprinting, including handheld strategies for wound dressing [2], portable systems across tissue-regeneration applications [13], and broader intraoperative and in situ bioprinting approaches [6,16]. More recent reviews have expanded this framework to skin-specific in situ bioprinting and contemporary handheld and robotic platforms [17,18,19]. However, the field has continued to evolve rapidly, with the emergence of SkinPen [10], Bioprint FirstAid [20], programmable smartphone-controlled handheld printing [9], and the BioGun platform evaluated in a porcine ischemic wound model [21], alongside the transition of robotic direct in situ skin bioprinting toward first-in-human evaluation [22]. These developments create a need for an updated assessment that not only describes handheld technologies but also distinguishes their current preclinical and clinical evidence from that of robotic in situ and closely related direct cell-delivery approaches and evaluates their readiness for clinical translation.

Accordingly, this review provides an updated, skin- and wound-focused translational assessment of portable handheld bioprinting. We integrate recent advances in device design and bioink requirements with clinical workflow, preclinical and human evidence, and regulatory and implementation challenges. Particular attention is given to the level of evidence supporting individual platforms and to distinguishing handheld bioprinting from robotic in situ systems and related direct cell-delivery technologies. By linking technological advances with the remaining evidence and implementation gaps, this review defines the current translational status of handheld skin bioprinting and the requirements for its further clinical development.

## 2. Methodology

### 2.1. Literature Search and Study Selection

This review was conducted as a structured narrative review focused on portable handheld and direct in situ bioprinting technologies, with particular emphasis on skin regeneration and wound treatment. The literature search covered publications available up to August 2026 and was performed using PubMed/MEDLINE, Scopus, Web of Science, and Google Scholar. Search terms included “handheld bioprinter”, “hand-held bioprinter”, “portable bioprinter”, “in situ bioprinting”, “point-of-care bioprinting”, “3D bioprinting”, “skin”, “wound”, “burn”, “skin regeneration”, and “bioink”, used individually and in relevant combinations. Additional searches using the names of individual bioprinting platforms were performed when required, and the reference lists of relevant publications were screened to identify additional studies.

Original experimental studies were prioritized for the assessment of device characteristics, bioink properties, preclinical efficacy, safety, and clinical outcomes. Review articles were used primarily to provide background information and to identify relevant primary studies. Publications were included if they addressed portable or handheld bioprinting, direct in situ deposition of cells or biomaterials, or technological aspects directly relevant to the development and clinical translation of such systems. Studies involving conventional or non-handheld bioprinting were also considered when they provided relevant information on printing mechanisms, bioink properties, or technological limitations. Publications unrelated to the technological, biological, preclinical, clinical, regulatory, or translational scope of the review were excluded.

### 2.2. Identification of Clinical Studies

Clinical studies were identified through searches of publicly available clinical trial registries, including ClinicalTrials.gov and the Australian New Zealand Clinical Trials Registry (ANZCTR), using terms related to bioprinting, skin regeneration, wounds, and burns. Studies involving human participants were considered when they evaluated bioprinted constructs or closely related direct in situ cell-delivery approaches relevant to skin regeneration. Therapeutic studies, in which the intervention was applied directly to patients, and studies using human-derived tissue for ex vivo bioprinting were distinguished during the analysis. Information from trial registries was supplemented with published study reports where available.

### 2.3. Assessment of the Current Market Landscape

The market analysis was conducted on a global scale and included commercially available devices and research-stage prototypes described up to 2026. Scientific publications, clinical trial records, and publicly available information from manufacturers and research organizations were considered. Devices were included if they were portable or handheld and intended for the direct in situ application of bioinks, cells, hydrogels, or other biomaterials for wound treatment or tissue regeneration. Related portable technologies were also considered when they represented clinically relevant alternatives to handheld bioprinting. Commercial and institutional sources were used primarily to determine product availability, technical characteristics, and development status, whereas conclusions regarding biological effects, safety, and therapeutic efficacy were based on scientific publications.

## 3. Portable Handheld Bioprinters

### 3.1. The Operating Mechanisms of Handheld Bioprinters

The core concept of portable handheld bioprinters lies in the controlled, metered deposition of bioink onto a surface. This precise delivery enables the efficient fabrication of customized skin substitutes directly at the injury site, accelerating healing and minimizing scarring. Two main types of handheld bioprinters exist: extrusion-based and inkjet-based ones (Table 1). Hybrid handheld systems may additionally combine extrusion with spray-based delivery or electrospinning as complementary deposition modes. Additionally, such systems can combine other approaches to orchestrate skin reparative processes, e.g., photobiomodulation [21,23].

Extrusion-based systems can be regarded as a “syringe with a precisely controlled plunger.” These systems use pneumatic or mechanical forces to extrude bioink through a nozzle, depositing it as continuous filaments [24,25]. The main advantage of extrusion-based bioprinting is its compatibility with a broad range of biomaterials, including highly viscous and cell-dense bioinks, allowing the fabrication of stable multilayer constructs [26]. However, the pressure required to force bioink through the nozzle exposes cells to shear stress, which generally increases with bioink viscosity and decreasing nozzle diameter and may consequently reduce post-printing cell viability [27,28]. These systems are widely used due to their relatively simple design and enable a wound defect to be filled according to a predefined 3D architecture. In handheld bioprinters for skin wounds, the operator activates the extrusion mechanism and controls the filling of the wound defect by manually positioning and moving the printing head [2,9]. For example, Hakimi et al. developed a battery-powered portable skin printer that extrudes a fibrin-based hydrogel in situ. Their bioprinter delivered two formulations (fibrinogen-hyaluronic acid and a thrombin crosslinker) through a microfluidic printhead to form a sheet matching the wound geometry. Although the printed sheet did not significantly enhance granulation or re-epithelialization compared to controls, it provided a biocompatible wound dressing without disrupting native healing [12]. Wang et al. described a programmable handheld bioprinter that uses a smartphone-controlled extrusion system to deposit bioinks with high precision. The system enables extremely rapid wound dressing and allows the composition and structure of the dressing to be tailored to the specific requirements of different wound types [9]. The SkinPen, designed by Zhou et al., represents a notable advance in in situ bioprinting, precisely depositing bioinks onto irregular wound surfaces. The combination of ultrasound and UV light promotes rapid gelation and superior bioadhesion, making it highly effective for wound stabilization. This sequential approach produced over three times stronger gel adhesion to the wound, addressing the common challenge of poor graft adherence [10]. Such devices are particularly useful for chronic wounds, such as diabetic ulcers, because they enable the fabrication of multilayered constructs that promote revascularization and re-epithelialization. The main disadvantages of extrusion systems are their lower resolution compared to some other modalities and their limited ability to print complex tissue structures [1,10]. Additionally, Tianyuan et al. developed an upgraded device that integrates spraying and electrospinning [11]. This design aims to address several wound-healing requirements simultaneously: the fibers provide hemostasis and structural support, the hydrogel delivers cells and hydration, and the spray supplies antibacterial agents or growth factors [11].

Inkjet-based systems offer higher precision than extrusion-based ones and are therefore well suited for applications requiring fine structural detail. This precision is achieved by propelling bioink droplets via thermal or piezoelectric forces, enabling high-resolution deposition. An example is the thermal inkjet bioprinter developed by Cui et al., which demonstrates high-precision deposition of biomaterials in controlled patterns. This system provides great versatility, allowing the fabrication of fine structures for soft tissue engineering while maintaining scalability for larger constructs [29]. However, the reliance on low-viscosity bioinks limits versatility, as many higher-viscosity bioinks cannot be used with inkjet printing [30,31]. Furthermore, the thermal and mechanical stresses (from piezoelectric or thermal actuators) imposed during droplet formation can compromise cell viability, particularly for sensitive primary cells or stem cells, often resulting in lower post-printing viability compared to extrusion methods. The drop-on-demand nature of inkjet technology also presents challenges in achieving uniform layer-by-layer deposition and high-aspect-ratio structures, as the small droplet volume and splashing can compromise deposition uniformity. While the periodontal bioprinter by Zhao et al. demonstrated the potential of this mechanism, scaling it for larger clinical wounds remains a challenge due to lower deposition rates [31]. Therefore, while inkjet systems are powerful for detailed, low-throughput applications, these inherent limitations in material compatibility, cell survival, and scalability must be overcome for them to become a frontline technology in in situ skin regeneration.

Compared with extrusion- and inkjet-based systems, laser-based bioprinting uses a short laser pulse to locally heat an absorptive layer, generating a vapor bubble whose expansion ejects a microdroplet of the adjacent cell-laden bioink onto the receiving substrate without passage through a nozzle [32,33,34]. The major advantage of laser-based bioprinting is therefore its nozzle-free mechanism, which eliminates nozzle clogging and nozzle-induced shear stress and enables high-resolution deposition of bioinks with high cell densities while maintaining high post-printing cell viability [32]. However, the technique requires precise control of laser–bioink interactions together with dedicated donor substrates and optical components, while inappropriate laser parameters may introduce thermal or mechanical stresses that adversely affect cells and deposition reproducibility [35,36]. These requirements substantially increase hardware complexity and make conventional laser-based systems difficult to miniaturize for handheld use [16]. Nevertheless, several studies have shown promising results for this technology in skin reconstruction [37,38]. Researchers acknowledge that handheld bioprinters are far simpler and more field-deployable than laser-based rigs [16]; therefore, laser bioprinting currently remains a laboratory or benchtop technique.

**Table 1:** Examples of portable hand-held bioprinters described in the literature.

Device Name	Mechanism	Printing Parameters			Bioink			Post-Printing Viability	In Vivo Model (If Reported)	Development Status, TRL	Target Application	Ref.
		Resolution	Pressure	Nozzle Diameter	Hydrogel	CL	Cell Type					
Handheld Skin Printer	Extrusion	~200–500 µm	10–30 psi	~22 G	Fibrin hyaluronic acid	Chemical	Mesenchymal stem cells	Not reported	Porcine full-thickness burn wounds	Prototype (preclinical), 4–5	In situ wound dressing for large burn injuries	Hakimi et al., 2018 [12] Cheng et al., 2020 [39]
Open-Source Handheld Bioprinter	Extrusion	~100–300 µm	<0.5 MPa	21–26 G	GelMA polyethylene oxide (emulsion porogen)	UV light	Fibroblasts Endothelial cells	>85%	N/A	Prototype (research), 3–4	In situ wound dressing	Ying et al., 2020 [3]
Bioprint FirstAid	Extrusion	~100–500 µm	Manual	Customizable	Two-component rapid-gelling hydrogel (e.g., alginate-based or fibrin-based)	Chemical	Autologous skin cells	>90%	N/A	Prototype (tech demonstrator), 6–7	First-aid in situ skin patch for superficial wounds Emergency use in space and remote environments	Warth et al., 2024 [20]
Smartphone-Controlled Handheld Bioprinter	Extrusion	Variable	Variable	Customizable	Hyaluronic acid-based hydrogel	Variable (Chemical, UV light)	HUVECs	>80%	Rodent full-thickness skin wounds	Prototype (preclinical), 4–5	In situ dressing of skin wounds	Wang et al., 2024 [9]
BioPen Handheld Bioprinter	Extrusion	Variable	30–300 kPa	18–21 G	Photocurable hydrogel (e.g., GelMA, methacrylated hyaluronan, chitosan, gellan gum, etc.)	UV light	hADSCs	>95%	N/A	Marketed (research use only), 5	Direct bioprinting onto injured tissues	AdBioInk BioPen product sheet (2020) [40]
SkinGun & CellMist System	Spray-based cell deposition	>1000 µm (spray)	N/A (spray)	N/A	CellMist™ media	None	Autologous skin stem cells	Not reported	Clinical trial (status unknown)	Preclinical/Clinical (FDA IDE approved in 2020 (not yet commercially available)), 6–7	Rapid treatment of burns and wounds by spraying patient’s own skin cells onto injuries	RenovaCare SkinGun (Clinical Trial Update, 2022) [41]
Handheld Multi-Mode Bioprinter	Hybrid: extrusion, spray, electrospin	~100–1000 µm	Not reported	22–26 G	PCL PEGDA Gelatin	UV light/Physical (electrospinning)	None	Not reported	N/A (in vitro only)	Prototype (preclinical), 4–5	In situ multi-layer wound dressing (sealant)	Tianyuan et al., 2021 [11]
SkinPen Handheld Bioprinter	Extrusion	Variable	30–80 kPa	22–25 G	GelMA Cu-BGn bioactive glass	UV light	Fibroblasts HUVECs	Not reported	STZ-diabetic rat wound model (infected ulcers)	Prototype (preclinical), 4–5	In situ wound healing for chronic/infected wounds	Zhou et al., 2023 [10]
Periodontal Bioprinter	Inkjet	<100 µm	N/A (piezo)	N/A	Fibrinogen Collagen	Chemical	hDPSCs HUVECs	Not reported	N/A (ex vivo tooth model only)	Prototype (preclinical), 3–4	In situ dental pulp regeneration	Zhao et al., 2024 [30] Duarte-Campos et al., [31]
BioGun (Biogan)	Extrusion	~300–500 µm	20–50 kPa	21–23 G	Fibrin Gelatin PEG	Chemical	MSCs	>85%	Porcine ishemic full-thickness skin wounds	Prototype (preclinical), 4–5	In situ wound healing	Revokatova et al., 2026 [21]

Abbreviations: CL—Crosslinking; Cu-BGn—copper-doped bioactive glass nanoparticles; DCM—dichloromethane; DFM—dynamic fluorescence microscopy; EPCs—endothelial progenitor cells; GelMA—gelatin methacrylate; HAMa—hyaluronic acid methacrylate; HUVECs—human umbilical vein endothelial cells; hADSCs—human adipose-derived stem cells; hDPSCs—human dental pulp stem cells; L929—L929 murine fibroblast cell line; MSCs—mesenchymal stromal cells; N/A—not applicable; PCL—polycaprolactone; PEG—poly(ethylene glycol); PEGDA—poly(ethylene glycol) diacrylate; PEO—polyethylene oxide; UV—ultraviolet.

### 3.2. Bioinks

Bioinks applied in bioprinting (including using handheld portable devices) are complex formulations composed of biopolymers, cells, and additional components that provide optimal conditions for both the printing process and subsequent cellular activity (Table 2). The bioink composition may include natural polymers such as collagen, alginate, and hyaluronic acid, as well as synthetic polymers such as polyethylene glycol (PEG). Their biodegradability can be tailored to match the rate of tissue regeneration, ensuring minimal inflammatory response and effective integration with host tissues. The physicochemical characteristics of bioinks (such as viscosity, elastic modulus, gelation rate, cohesion, and bioadhesion) play an important role. Optimal viscosity ensures accuracy and stability during extrusion, while the mechanical strength and elasticity of the resulting gel affect the structural integrity of the printed constructs. Moreover, bioinks must be biocompatible, supporting cell adhesion, proliferation, and differentiation without inducing cytotoxicity or triggering an immune response.

Key rheological parameters such as viscosity, shear-thinning behavior, storage modulus (G′), and loss modulus (G″) are critical for both the extrusion process and the post-printing stability. For clinical application, an optimal bioink should exhibit shear-thinning behavior, where its viscosity decreases under shear stress (e.g., during extrusion through a nozzle) and recovers rapidly to a stable gel state after deposition. A typical bioink displays a zero-shear viscosity ranging from 10^4^ to 10^6^ cP to allow for sufficient shape retention, while a yield stress (the minimum force required to initiate flow) of 50–200 Pa is often desirable to prevent sagging and spreading post-deposition [42,43]. The storage modulus (G′), which represents the elastic behavior, provides a measure of the hydrogel’s mechanical stiffness and its ability to resist deformation. For skin regeneration, G′ values of printed hydrogels are often reported in the range of 100 to 10,000 Pa, mimicking the mechanical properties of native dermal tissue (which can range from 5 to 100 kPa). For instance, while fibrin-based hydrogels have a G′ of 100–1000 Pa, providing a soft, cell-friendly environment, they may lack the mechanical integrity for maintaining 3D architecture under pressure [12,44]. More robust systems, such as those using GelMA or alginate, often exhibit G′ values of 1000–10,000 Pa, promoting better structural stability during handling [45]. Crosslinking strategies further modulate these values: photocrosslinked GelMA can significantly increase G′ depending on the UV exposure time and photoinitiator concentration, impacting the cellular microenvironment. A quantitative comparison of these parameters is essential for system optimization, allowing researchers to predict print fidelity and tissue integration. For example, a highly viscous, cohesive bioink may exhibit excellent structural fidelity but poor bioadhesion, whereas a low-viscosity bioink will flow easily but struggle to maintain the desired shape. Ultimately, bioink formulation must achieve a balance between these rheological properties to ensure both the printability of the device and the functional performance of the final tissue construct.

Quantitative comparison across bioink systems highlights distinct rheological profiles relevant to handheld bioprinting. Fibrin-based hydrogels exhibit G′ values of 100–1000 Pa and low viscosities (0.1–1 Pa·s), providing excellent biocompatibility but limited shape fidelity [12,44]. In contrast, alginate (G′: 1000–5000 Pa) and GelMA (G′: 1000–10,000 Pa) offer greater mechanical stability, with shear-thinning indices of 0.15–0.30 facilitating smooth extrusion while maintaining post-deposition integrity [45,46]. The yield stress, typically ranging from 20 to 200 Pa for printable formulations, is a critical predictor of shape retention, with values below 10 Pa leading to spreading and values above 500 Pa compromising extrudability and cell viability [27,28]. An optimal handheld bioink should therefore balance these parameters, in particular a zero-shear viscosity of 10–100 Pa·s, a shear-thinning index of 0.1–0.3, a yield stress of 20–200 Pa, and a G′ of 1–10 kPa, to ensure both printability and functional performance. However, variability in measurement conditions across studies underscores the need for standardized rheological characterization protocols to enable meaningful cross-study comparison and accelerate clinical translation.

Bioadhesion/cohesion is especially critical for bioinks designed for use in handheld portable devices. The bioadhesive properties arise from specific functional groups, such as carboxyl (–COOH), amine (–NH_2_), and hydroxyl (–OH) groups, which engage in covalent and non-covalent interactions (e.g., hydrogen bonding, electrostatic forces) with the tissue surface [47]. These adhesive properties can be additionally modulated by incorporating biomimetic molecules, such as arginine-glycine-aspartate (RGD) motifs, which promote cell attachment and tissue integration [48,49]. Cohesion, in turn, determines the internal strength and mechanical stability of the bioink, ensuring that printed constructs maintain their structural integrity after deposition. This parameter depends on the viscosity, elastic moduli, and mechanical properties of the hydrogels employed. An optimal balance between cohesion and bioadhesion is necessary to prevent the deformation and collapse of printed structures and to maintain their spatial organization. For example, fine-tuning the degree of gelation and crosslinking allows bioinks to achieve the desired mechanical properties, enhancing both their stability and controllability during printing [46,50,51,52].

As discussed above, incorporating RGD-modified bioinks and functionalized hydrogel systems during bioprinting significantly improves cell adhesion, proliferation, and tissue integration by enhancing interactions with integrin receptors. RGD-functionalized alginate hydrogels employed in FRESH bioprinting have demonstrated superior cell attachment and post-printing viability, thereby ensuring the stability of the printed constructs [53]. Similarly, hydrogels bearing carboxyl (–COOH), amine (–NH_2_), and hydroxyl (–OH) groups have been developed to mimic the native extracellular matrix environment, resulting in enhanced biomechanical properties and cellular responses [54]. Tissue-specific bioinks have further optimized the cellular microenvironment, enabling improved spatial organization and long-term stability of constructs in bioprinted skin [55]. Collectively, these advances ensure better biocompatibility, mechanical integrity, and functionality.

The Laser Skin Substitute Bioprinter (LSSB), for instance, has been used to print alginate–fibrin bioinks directly onto wounds, leading to improved fibroblast proliferation and angiogenesis—two essential processes for wound healing and tissue vascularization. Alginate provides structural stability and rapid gelation, which are particularly valuable in handheld and in situ bioprinting applications, while fibrin stimulates cellular activity and tissue integration [37,46]. Other handheld bioprinting systems, such as the Handheld Skin Printer, employ a microfluidic cartridge that deposits biocompatible hydrogel sheets composed of alginate and fibrin, which rapidly crosslink into structured skin-like matrices. This approach has demonstrated improved adhesion to wound surfaces and helps maintain a stable environment that supports dermal and epidermal cell proliferation, thus greatly enhancing the regenerative potential of the printed tissues [12]. In a similar vein, handheld bioprinting holds considerable promise for de novo vascular formation through the direct deposition of vascular bioinks comprising endothelial and smooth muscle cells, enabling in situ blood vessel regeneration—a critical step toward large-scale tissue engineering [2,31]. In the context of portable bioprinting, new bioink formulations have also been explored, including chitosan-kaolin nanocomposite hydrogels with enhanced mechanical stability and controlled degradation. These hydrogels exhibit excellent cohesive strength and a homogeneous cell distribution, making them suitable for wound repair and skin grafting [56].

**Table 2:** Examples of bioink formulations and their characteristics.

Bioink Composition	Printing Context	Cell Type	CL	Relevant Properties	Experimental Model	Advantages	Limitations	Ref.
Alginate, collagen; fibrinogen, HA, collagen	Handheld in situ bioprinting	FB KC	Ca^2+^-mediated; fibrinogenesis; gelation	Rapid sheet formation Controlled sheet morphology Uniform cell distribution Deposition rate 0.3–1.6 cm^2^/s	In vitro; murine excisional wounds Porcine full-thickness wounds	Rapid direct deposition Local control of material and cell distribution Conformal sheet formation	Proof-of-concept; No significant improvement in re-epithelialization vs. control in porcine study	Hakimi et al., 2018 [12]
Agarose; collagen I	Handheld in situ bioprinting	hDPC EC	Thermal gelation	Rapid gelation Structural stability Maintenance of original shape Vascular tube formation	In vitro Ex vivo root canals	Suitable rheology for handheld deposition Shape retention Supports vasculogenesis	Ex vivo proof-of-concept Further in vivo validation required	Duarte Campos et al., 2020 [31]
Alginate; chitosan; kaolin nanoclay	Handheld in situ bioprinting	OB FB	polyelectrolyte complexing; H bonding; Ca^2+^-mediated	Improved mechanical properties and self-standing printability Homogeneous component and cell distribution	In vitro	Homogeneous in situ mixing Improved structural stability Good post-printing shape fidelity	In vitro validation only No in vivo wound-healing model	Bhattacharyya et al., 2023 [56]
Alginate; RGD-modified alginate	FRESH bioprinting (non-handheld)	ahDFB	Ca^2+^-mediated	High shape fidelity High initial cell viability RGD formulations supported prolonged cell viability	In vitro	RGD-mediated cell attachment Tunable physical properties Stable printed geometry	In vitro only Printing accuracy dependent on formulation	Zhu et al., 2022 [53]
Thiolated HA; thiolated gelatin; PEG acrylate and PEG alkyne crosslinkers; optional tissue-derived ECM; unmodified HA and gelatin	Extrusion bioprinting (non-handheld)	phHep liver spheroid	Thiol-acrylate crosslinking	Tunable shear stiffness (~100 Pa–20 kPa) Soft material during extrusion Increased stiffness after secondary crosslinking	In vitro	Broadly tunable mechanical properties Tissue-specific biochemical composition Improved extrusion by unmodified HA/gelatin	Multicomponent formulation Demonstrated in conventional rather than handheld bioprinting	Skardal et al., 2015 [54]
HA/gelatin hydrogel; liver ECM; PEG-based crosslinkers	Extrusion bioprinting (non-handheld)	liver spheroid	PEG-based crosslinking UV crosslinking	Tunable stiffness High cell viability Measurable albumin and urea production	In vitro	Tissue-specific biochemical signals Controllable mechanical properties Post-print stabilization	In vitro only Not approved for handheld/in situ use	Skardal et al., 2016 [55]
Cell suspension deposited onto Matriderm	Laser-assisted bioprinting (non-handheld)	FB KC	N/A	Precise spatial positioning of cell types Multilayered epidermis formation Collagen production by fibroblasts	Nude mouse dorsal skin-fold chamber Full-thickness skin wound	Precise organization of multiple cell types Skin-like tissue formation in vivo	Requires specific infrastructure Early epidermal differentiation after 11 days Not a handheld approach	Michael et al., 2013 [37]

Abbreviations: ahDFB—adult human dermal fibroblasts; CL—Crosslinking; EC—endothelial cells; FB—fiibroblasts; HA—hyaluronic acid; hDPC—human dental pulp cells; KC—keratinocytes; N/A—not available; OB—osteoblasts; phHep—primary human hepatocytes.

## 4. Clinical Applications and Workflow

**Figure 1:**
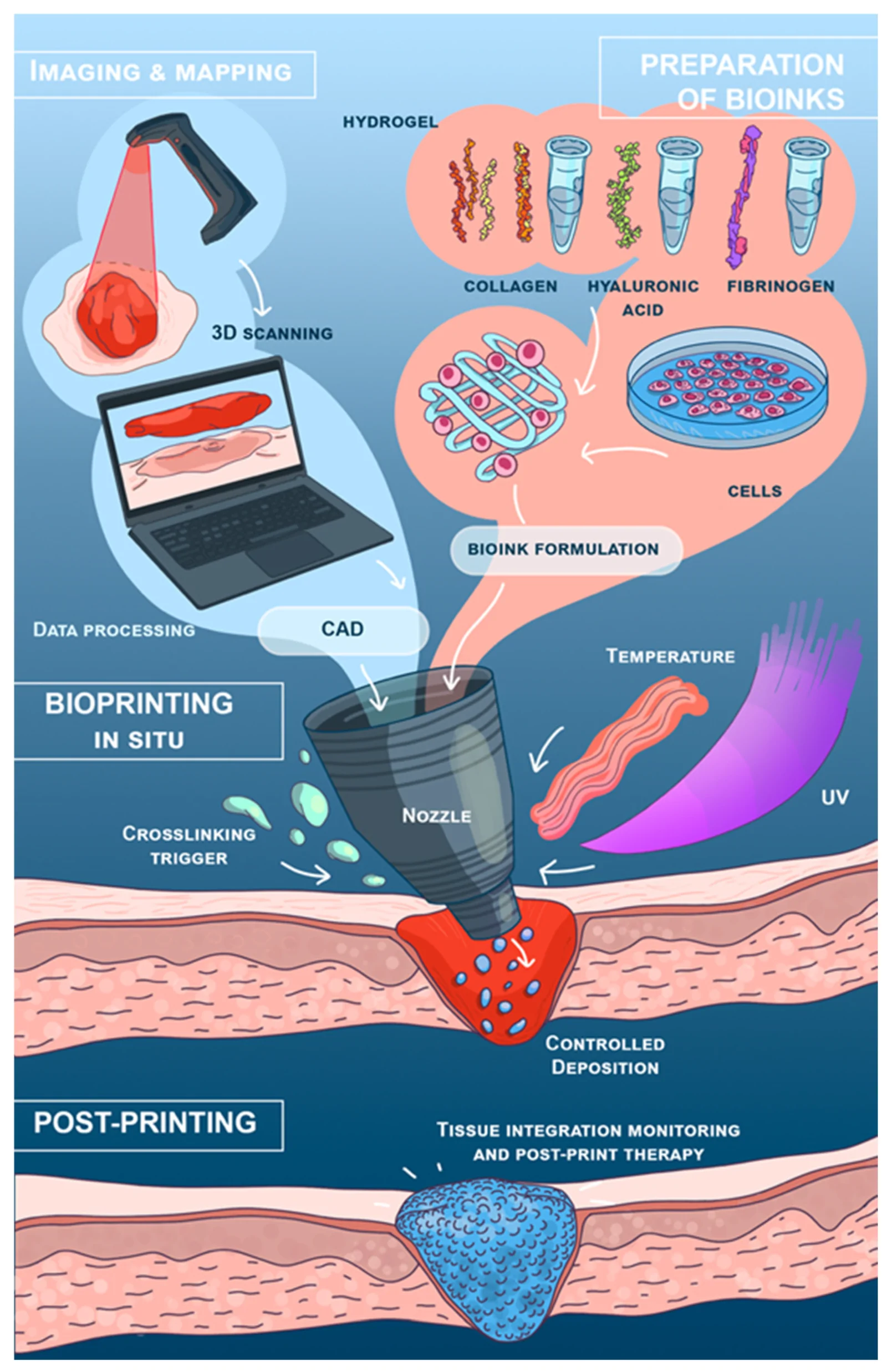
Workflow of a portable handheld bioprinting process for skin regeneration. The workflow includes wound assessment and planning (3D scanning and CAD-based mapping), bioink preparation, layer-by-layer in situ deposition with crosslinking, and post-printing integration and monitoring.

Portable bioprinters integrate several enabling technologies into a seamless workflow that permits real-time treatment of wounds. Imaging modalities such as 3D scanning allow for the accurate mapping of wound geometry, which is essential for precise bioink deposition. This is particularly useful for irregular or deep wounds where conventional dressings may prove inadequate. An overview of this end-to-end workflow is illustrated in Figure 1.

The practical relevance of different printing mechanisms depends not only on their nominal printing resolution but also on the requirements of the target wound and the mode of device operation. In manually operated handheld systems, the printhead is positioned and moved by the operator; consequently, for large-area or predominantly planar skin defects, where the primary objective is conformal coverage rather than reconstruction of microscale tissue architecture, very high intrinsic printing resolution may be less decisive [9,12]. In this context, extrusion-based systems provide a practical balance between controlled deposition, compatibility with cell-laden hydrogels, and the formation of continuous wound-conforming layers, which may partly explain the predominance of extrusion-based designs among handheld bioprinters developed for skin applications [9,12,39]. By contrast, droplet-based deposition offers greater control over the spatial positioning of cells and biomaterials and may therefore be advantageous when localized or sequential deposition is required [1]. Spray-based deposition favors rapid coverage of large and irregular surfaces but provides less control over spatial organization, whereas electrospinning is primarily suited to the formation of conformal fibrous layers rather than precisely organized three-dimensional cellular constructs [57,58,59].

Automation further enhances device functionality: the regulation of nozzle pressure and extrusion speed ensures uniform layer deposition, even on geometrically complex wound surfaces. The handheld Multi-Mode Bioprinter developed by Tianyuan et al. illustrates how a single device can integrate as many as three printing techniques—extrusion, spray, and electrospinning [11]. Such hybrid bioprinting broadens the clinical applicability of the technology, extending its utility from emergency medicine to reconstructive surgery.

The workflow of handheld bioprinters encompasses advanced imaging, precise bioink preparation, and real-time printing, thereby simplifying wound treatment. For instance, the In Situ Bioprinter incorporates an integrated 3D scanning tool that generates high-resolution morphometric maps of the wound, ensuring that bioink deposition conforms precisely to wound geometry and depth [1]. Automated features, including pressure control and nozzle manipulation, help reduce layer-to-layer variability and preserve construct homogeneity even on complex topographies. Despite these automated capabilities, the clinician’s role remains essential in guiding the process. Devices such as the SkinPen combine automated printing with manual adjustments, enabling real-time decision-making based on wound condition [10]. The clinician can, for example, ensure that the composition and placement of the bioink meet the patient’s specific needs while the device is in operation. This hybrid approach balances precision with adaptability, making portable bioprinters well suited to dynamic clinical environments.

Portable, handheld bioprinters show considerable promise in delivering effective, precise treatments for both acute and chronic wounds, underscoring their potential transformative role. Continued advances in bioink formulations, device miniaturization, and automation will allow these systems to find broader application and may eventually establish them as a staple in modern healthcare.

### 4.1. Clinical Studies

Clinical trials involving bioprinting techniques remain scarce, and most are in early phases—Phase I or II. The majority of these studies focus on the treatment of severe burns, chronic ulcers, and applications in reconstructive surgery. Bioprinting offers the potential for patient-specific tissue engineering, with advantages over conventional autografting, including reduced donor-site morbidity and improved graft integration. However, few approaches have progressed to large-scale human testing.

In 2025, Briones et al. reviewed clinical trials on bioprinting-based skin regeneration registered in publicly accessible databases [60] and identified several trends among active studies. One relevant study (NCT04925323; BIOPSKIN) focuses on generating GMP-compliant validation batches of bioprinted dermo-epidermal substitutes from human surgical tissue. A further search for ongoing clinical trials involving bioprinting identified the studies summarized in Table 3.

Long-term follow-up data on graft performance in human patients remain poorly documented. Although early results indicate accelerated wound healing, the long-term functionality of bioprinted tissues (such as elasticity, durability, and response to environmental stressors) is not yet known [60]. Many studies focus on short-term outcomes, including re-epithelialization rates and inflammatory responses, while longer observation periods are needed to assess graft viability, as well as the prevention of fibrosis or contracture.

#### 4.1.1. Therapeutic Clinical Studies

Human clinical evidence for handheld and direct in situ skin bioprinting remains very limited. Among the studies retained in Table 3, NCT04890574 evaluates the CellMist™ System for the treatment of deep second-degree burns using the SkinGun™ device (RenovaCare, Inc., Scottsdale, AZ, USA). This is a prospective, multicenter, single-arm, open-label feasibility study involving 14 adults with deep second-degree burns. The study primarily focuses on safety, including adverse events and the need for secondary surgical interventions during 12 months of follow-up, while wound healing and pain are also monitored. However, the SkinGun™ performs spray-based cell deposition rather than spatially controlled layer-by-layer 3D bioprinting and therefore represents a handheld direct cell-delivery approach closely related to bioprinting. The small planned sample size, absence of randomization and a comparator group, and lack of publicly posted study results substantially limit conclusions regarding therapeutic efficacy.

ACTRN12625000088448 (LIGO-SKIN-A001) represents a direct clinical application of in situ skin bioprinting. This first-in-human, single-arm, open-label study evaluates the robotic Ligo In situ Bioprinting System, which deposits autologous skin cells and a two-component biomaterial directly into the wound. Up to 10 participants were planned, with safety as the primary endpoint and time to wound closure as the secondary endpoint. A 2026 conference report described the initial clinical study and preparation of a subsequent phase [22]. However, the small uncontrolled cohort and limited outcome data restrict extrapolation to complex burn injuries.

Clinical evaluation of handheld and in situ skin bioprinters is still at an early stage, with only limited preliminary results available. Thus, the evidence base is only beginning to emerge, while robust assessment of clinical efficacy and meaningful comparisons between different platforms remain subjects for future studies.

#### 4.1.2. Observational and Ex Vivo Studies

NCT04925323 (BIOPSKIN) focuses on the generation of GMP-compliant validation batches of a bioprinted dermo-epidermal skin substitute from human surgical tissue rather than on a therapeutic intervention. Human participants therefore serve only as tissue donors, and the bioprinted construct is not applied clinically. The study primarily addresses manufacturing standardization and GMP compatibility and does not provide direct evidence of therapeutic efficacy or clinical safety.

### 4.2. Preclinical and Clinical Evidence

#### 4.2.1. Human Studies

Although still in the early stages, clinical trials on bioprinted skin have already yielded promising results. Recent studies have shown that bioprinted skin grafts can improve wound healing compared with traditional autografts.

Initial clinical data for bioprinted skin substitutes are beginning to emerge. A recent trial compared StrataGraft—a bioengineered allogeneic cellularized skin construct (which can be produced via bioprinting)—with conventional autografts in patients with deep partial-thickness burns. Patients treated with StrataGraft spent an average of 4.8 fewer days in the hospital than those receiving traditional autologous skin grafts (17.7 vs. 22.5 days). This reduction in hospital stay may reflect faster wound healing or fewer complications, potentially reducing the burden on healthcare providers and improving patient recovery. These clinical findings support the potential of bioprinted skin grafts to enhance treatment efficacy and outcomes in burn management. Given the mechanistic similarities, bioprinted grafts may confer comparable advantages, particularly in terms of faster revascularization and better graft integration. However, additional clinical trials are needed to confirm these assumptions and to quantify the specific impact of bioprinted skin constructs on length of hospital stay and post-surgical recovery [61].

Health economic evaluations remain limited; however, several reports suggest that despite higher initial production costs, bioprinted skin substitutes may offer long-term economic advantages by reducing operative time, the need for postsurgical interventions, and the duration of hospitalization [14,62,63]. A recent scoping review of clinical and observational bioprinting trials identified 11 eligible studies: seven involved in vitro patient-derived models and four were interventional trials aiming to implant bioprinted tissue [60]. Although long-term outcomes are still being evaluated, early patient responses indicate improved graft engraftment, lower complication rates, and acceptable recovery pathways. As clinical evidence continues to accumulate, bioprinting may eventually become a core technique in personalized wound reconstruction and complex reconstructive surgery. As clinical translation advances, it remains to be seen whether similar improvements in tissue quality will be observed in patients [60].

#### 4.2.2. Animal Studies

Preclinical studies have also provided evidence of improved healing quality with bioprinted skin constructs. Jorgensen et al. (2023) developed full-thickness human skin equivalents containing epidermal, dermal, and hypodermal layers and evaluated them in vivo [64]. After transplantation of the bioprinted skin into full-thickness wounds, more organized collagen deposition was observed, along with an epidermal structure resembling normal skin, including the formation of dermal–epidermal ridges [64]. The researchers noted reduced scarring and contraction: the bioprinted graft supported a normal collagen architecture with minimal fibrosis, in contrast to conventional grafts that often heal with scar tissue. The implanted human cells showed growth and integration into the wound bed, contributing to the formation of a well-organized epidermis [64]. These results indicate that bioprinted skin can regenerate more physiologically functional tissue than standard grafting techniques, although, to date, this has been demonstrated only in animal models (mouse and pig) rather than in human trials.

Notably, a large-scale GMP-compliant bioprinted skin equivalent (Poieskin^®^) achieved a mean graft take of 91.8% on day 16 in immunosuppressed mice, compared with 100% for human split-thickness skin graft controls, with no significant difference in graft take, supporting its technical feasibility for future clinical application [65].

Bioprinted grafts have also been associated with lower rates of inflammatory response, hypertrophic scarring, and secondary infection in immunocompromised or chronically wounded patients, although the majority of this evidence currently derives from preclinical investigations and case reports [1,2,39,63].

### 4.3. Regulatory Challenges

The regulatory landscape for bioprinted tissues remains complex and fragmented, as bioprinted skin constructs do not fit neatly into existing classification frameworks for either medical devices or biological therapies. Regulatory agencies such as the U.S. Food and Drug Administration (FDA) and the European Medicines Agency (EMA) require extensive preclinical and clinical validation before granting approval, yet standardized testing protocols specific to bioprinted grafts are still under development.

The FDA has issued guidance on “Additively Manufactured Medical Devices” (2017), which provides general recommendations for device design and manufacturing, but it is not specific to bioprinting [66]. The regulatory pathway for a bioprinted product depends on its composition, intended use, and mechanism of action. For combination products, assignment to a lead FDA center is based on the product’s primary mode of action (PMOA). If the device-related function provides the PMOA, primary review responsibility may fall to the Center for Devices and Radiological Health (CDRH); if the biological or cellular activity provides the PMOA, it may fall to the Center for Biologics Evaluation and Research (CBER), depending on the specific biological product. For combination products, the Office of Combination Products (OCP) coordinates the assignment of the appropriate lead FDA center [67]. For the CellMist™/SkinGun system, the reported Investigational Device Exemption (IDE) status indicates investigational use and should not be interpreted as marketing authorization or as establishing a specific Premarket Approval (PMA) or Biologics License Application (BLA) pathway.

In the European Union, regulatory classification depends on the intended purpose, composition, and mode of action of the complete product. A handheld bioprinter may be classified as a Class IIa or IIb medical device, depending on its intended purpose and specific characteristics; however, the applicable classification should be determined on a product-specific basis. Cell- or tissue-based products may qualify as advanced therapy medicinal products (ATMPs) when they meet the applicable criteria for somatic cell therapy or tissue-engineered medicinal products; however, the presence of cells alone is not sufficient for ATMP classification. If a medical device forms an integral part of an ATMP, the product may be classified as a combined ATMP [68].

The absence of specific International Organization for Standardization (ISO) and American Society for Testing and Materials (ASTM) standards for handheld bioprinting is a major barrier. While general standards exist for biocompatibility (ISO 10993), medical device quality management (ISO 13485), and cell-based products, there are no standards tailored to the unique challenges of in situ bioprinting.

Bioink quality control must encompass strict batch-to-batch reproducibility of physicochemical properties (e.g., viscosity, gelation time, and elastic modulus), sterility, and endotoxin levels. For clinical applications, bioinks must conform to Good Manufacturing Practice (GMP) guidelines, ensuring that every batch has consistent printability, cell compatibility, and in vivo performance. Regarding release criteria, the bioink should be designed to degrade and release its cellular and molecular cargo in a manner that aligns with the wound healing timeline. This involves controlled degradation kinetics: a slow degradation may hinder tissue ingrowth, while rapid degradation can lead to loss of structural support. The release of cells and growth factors should be spatially and temporally controlled to promote a continuous and coordinated healing process. Standardized in vitro assays for degradation, drug release, and cell viability, such as those defined in ISO 10993 for biological evaluation, are essential for establishing benchmark performance criteria. Furthermore, Kirillova et al. (2020) [15] emphasized the difficulty of establishing mechanical stability criteria for bioprinted grafts. Since bioprinted skin constructs must withstand physical stress following transplantation, rigorous mechanical testing protocols are necessary before clinical translation. Most current studies rely on short-term animal models, whereas human-scale mechanical performance data are required for regulatory approval [15].

Another major hurdle is the absence of universal safety standards for bioprinted skin substitutes. Unlike synthetic grafts, bioprinted constructs rely on living cells, which raises challenges related to sterility assurance, immune compatibility, and long-term performance evaluation. Mladenovska et al. (2023) identified poor bioink standardization as a key issue, noting that variations in cell source, hydrogel formulation, and crosslinking method can significantly affect the mechanical and biological properties of the final graft [14].

Ultimately, defining clear, measurable release and quality-control parameters for bioink is a prerequisite for regulatory approval and widespread clinical acceptance.

## 5. Current Market Landscape of Portable Handheld Bioprinters

The current market landscape was assessed on a global scale based on the scientific literature and publicly available information from manufacturers and research organizations included in this review. The analysis included commercially available devices and research-stage prototypes described up to 2026. Devices were included if they were portable or handheld and designed for the direct in situ application of bioinks, cells, hydrogels, or other biomaterials for wound treatment and tissue regeneration. Portable systems based on related technologies, such as electrospinning, were also considered when they were intended for direct wound treatment and represented a clinically relevant alternative to handheld bioprinting. Stationary bioprinters and robotic systems were not included in this section. The devices were divided into commercially available systems and research-stage prototypes according to the information provided in scientific publications, clinical trial records, and publicly available manufacturer or institutional sources.

### 5.1. Commercially Available Handheld Bioprinters

The handheld portable bioprinter market is still in its early stages, with few commercially available products and numerous prototypes under development.

One of the few commercially available handheld bioprinters is the AdBioInk BioPen-X (Advanced Bioink Technologies, Kocaeli, Turkey), an in situ tissue regeneration device [69]. The BioPen-X is an extrusion-based portable handheld bioprinter equipped with a bioink cartridge and an integrated light source for photocrosslinking (WO2023244197A1). Its printing speed and light source can be adjusted according to the printing conditions, allowing direct deposition of photocrosslinkable hydrogel formulations (WO2023244197A1). BioPen-X has also been experimentally used for the fabrication of GelMA-based hydrogel wound dressings (Istinye University MSc thesis, 2024). Additionally, the BioPen-X is more affordable than large-scale industrial bioprinters, offering promising potential for initial clinical use and research. However, scalability remains a concern, as current handheld devices are primarily designed for treating localized wounds rather than covering extensive tissue areas. Integrating such devices into routine hospital practice will require further advances in automation, bioink production, and regulatory approval [40].

Another noteworthy commercial product is the Spincare^®^ Wound Treatment System, a portable electrospinning device that deposits nanofiber scaffolds directly onto wounds. Unlike extrusion-based systems, Spincare^®^ employs a non-contact spinning process to create a biocompatible, skin-adherent matrix that facilitates re-epithelialization. In a prospective clinical study involving partial-thickness burns, Spincare^®^ achieved wound closure rates exceeding 97% by day 21, with no serious adverse events and high patient mobility post-application [70]. Its battery-powered operation, compact design, and ease of use by routine medical personnel make it well-suited for point-of-care wound management. Although Spincare^®^ does not deposit living cells or structured hydrogels, its clinical practicality and healing outcomes position it as a viable alternative to cell-based bioprinting approaches for superficial skin regeneration [71].

### 5.2. Prototypes and Research-Stage Handheld Bioprinters

The translational status of handheld bioprinters is at an early stage. Most systems (e.g., Hakimi et al. [12], Ying et al. [3], Tianyuan et al. [11], Revokatova et al. [21]) are still in the preclinical prototype stage, with their efficacy primarily validated in animal models (porcine, rodent) or in vitro. A crucial step forward has been the demonstration of clinical-grade systems, such as the smartphone-controlled printer by Wang et al. [9], which offers a balance of mobility and precision, and is currently being validated in preclinical settings. The SkinGun/CellMist system represents a translational milestone, having received FDA IDE approval for clinical trials, yet it is not commercially available. This suggests that while proof-of-concept is established, challenges in scaling, sterility, and regulatory compliance delay market entry.

**Figure 2:**
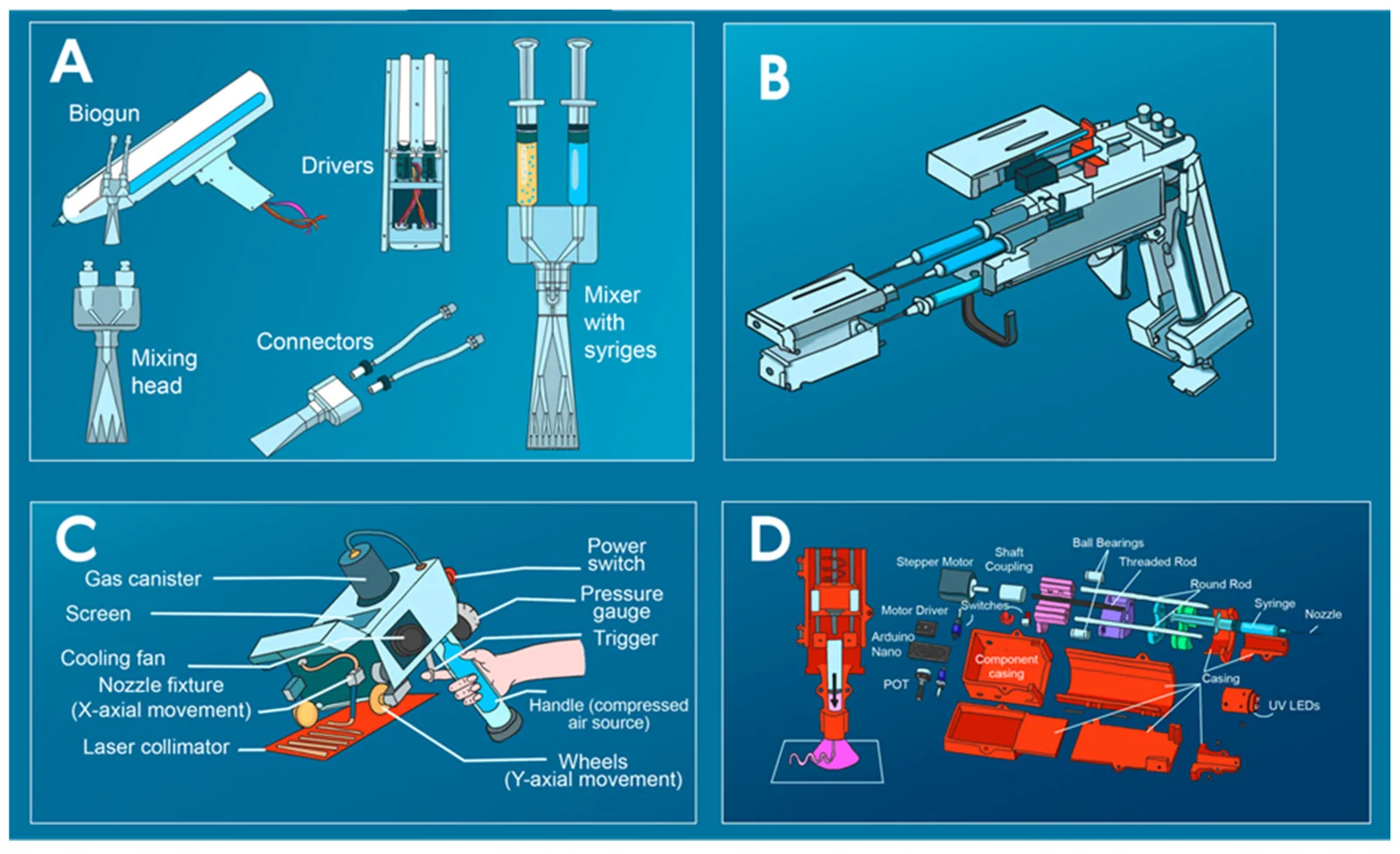
Schematic images of some portable handheld bioprinters: (**A**) BioGun by Revokatova et al. (2026) [21], CC BY 4.0. (**B**) Hybrid handheld bioprinter by Tianyuan et al. (2021) [11], CC BY 3.0. (**C**) Smartphone-operated handheld bioprinter by Wang et al. (2024) [9], CC BY 4.0. (**D**) Open source extrusion bioprinter by Ying et al. (2020) [3], CC BY-NC-ND 4.0.

The AdBioInk BioPen-X is a rare example of a commercially available device, but it is explicitly marketed for research use only. This highlights a critical bottleneck in the translation process: most prototypes do not meet the stringent requirements for a medical device [72]. The path to clinical adoption requires progression through several technological readiness levels (TRLs), from basic research (TRL 1–3) to proof-of-concept in relevant environments (TRL 4–6), and finally to demonstration and deployment in clinical settings (TRL 7–9). Currently, most handheld bioprinters are below TRL 6. Key hurdles to reaching TRL 7+ and clinical trials are the lack of standardized GMP-compliant bioink production, insufficient evidence of long-term construct integration and safety, and the need for robust, user-friendly device interfaces suitable for surgical environments. The transition from preclinical proof-of-concept to clinical reality requires a concerted focus on regulatory compliance, scalability, and economic viability. Representative examples of portable handheld bioprinters are shown in Figure 2.

### 5.3. Comparison of Handheld and Stationary Bioprinters

In the evolving clinical landscape and commercial market of bioprinting, it is natural to compare emerging handheld systems against the established paradigm of conventional stationary bioprinters. These two technological approaches can be viewed from two distinct perspectives. On the one hand, they represent alternative, competing strategies for tissue fabrication, each with its own trade-offs in precision, scalability, and cost. On the other hand, they are fundamentally designed to solve different clinical problems: stationary systems excel in the pre-operative, laboratory-based fabrication of complex, high-fidelity grafts, while handheld devices are optimized for real-time, point-of-care intervention in acute and emergency settings. Table 4 provides a dedicated comparison of these two classes of bioprinters across key parameters, including precision, scalability, portability, cost, and clinical applicability, to clarify their respective roles and guide technology selection based on the specific clinical scenario.

## 6. Challenges and Future Directions

Although 3D bioprinting holds great promise for the fabrication of complex biological structures, it faces a range of challenges that limit its broader applicability and utility. These challenges span multiple aspects of the bioprinting process, from material limitations to technical constraints in methodologies. The key challenges associated with 3D bioprinting are summarized in Table 5 [13,73,74,75,76,77].

Despite the formidable nature of these technical hurdles, ongoing research and technological innovation are steadily resolving them. Advances in 4D bioprinting, in which scaffolds are programmed to change shape in response to stimuli, together with the development of novel bioinks and printing strategies, offer promising avenues to overcome current limitations and extend the capabilities of 3D bioprinting [73,77].

Notwithstanding this potential, the widespread clinical introduction of portable bioprinting technologies is hindered by a distinct set of barriers, which are outlined in Table 6. Overcoming these multifaceted barriers is critical to the successful commercialization of portable bioprinters. A concerted effort among researchers, clinicians, regulators, and commercial partners is needed to address technological challenges, clarify regulatory pathways, reduce financial obstacles, facilitate integration into clinical workflows, and promote acceptance of this emerging regenerative medicine technology [6,63].

Despite recent technological progress, several important knowledge gaps remain before handheld bioprinting can be translated into routine clinical practice. Most importantly, controlled human studies of true handheld systems are lacking, and long-term functional outcomes of regenerated skin remain insufficiently characterized. Standardized criteria for handheld-specific bioink properties, printing performance, and operator-dependent reproducibility are also needed to enable meaningful comparison between platforms. In addition, sterility, GMP-compatible production, clinical workflow integration, regulatory pathways, and cost-effectiveness require further validation under clinically relevant conditions.

**Figure 3:**
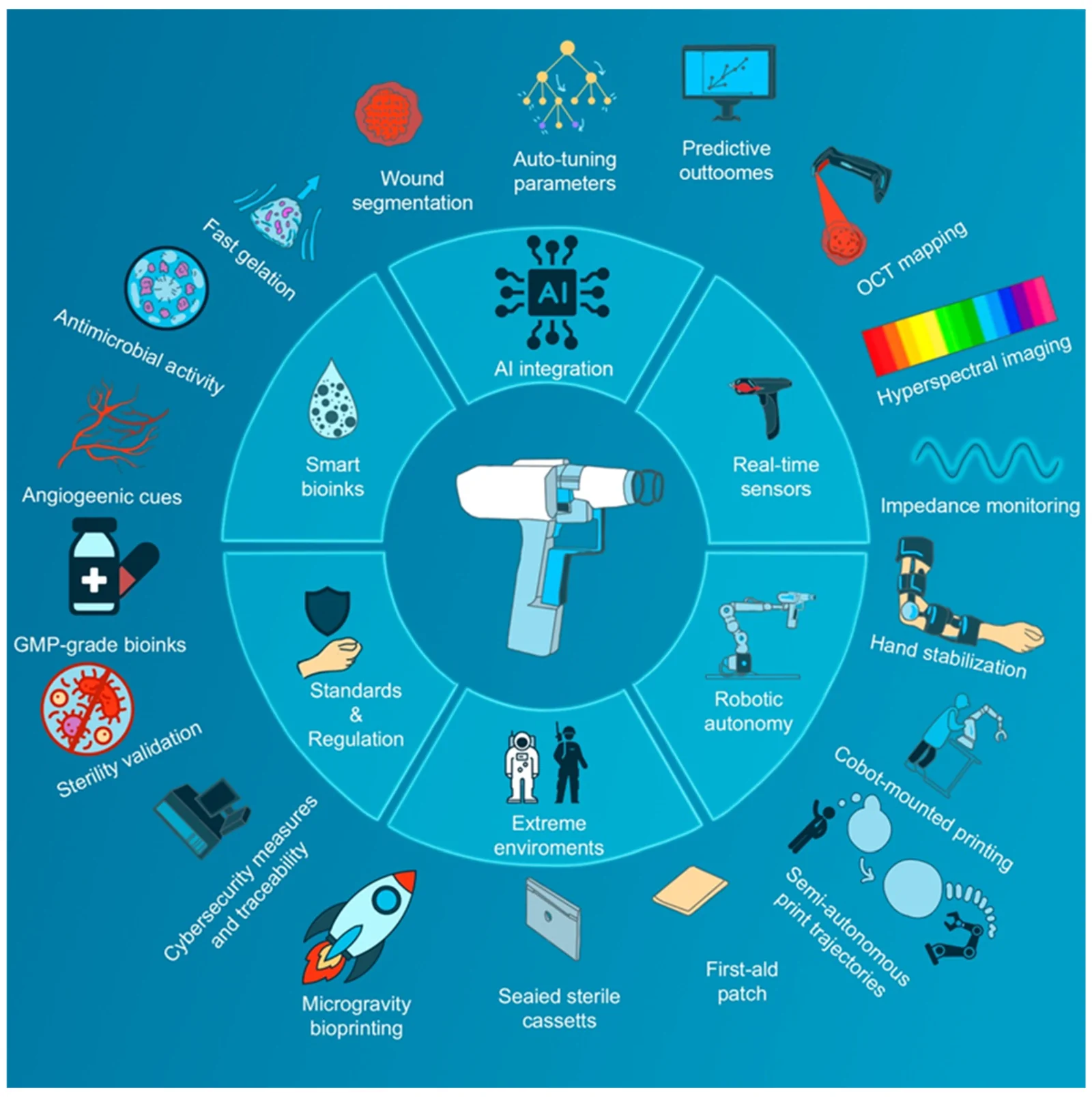
Convergence map for next-generation handheld bioprinting. The central node represents a portable extrusion-based bioprinter; the inner ring depicts six domains: smart bioinks, artificial intelligence, real-time sensors, robotic autonomy, space-readiness, and standards/regulation. The outer ring illustrates selected future innovations linked to each domain: (1) Smart bioinks (fast gelation promoted by special properties: stimuli-responsive, hemostatic/adhesive; antimicrobial and pro-angiogenic formulations; ECM-mimetic chemistries such as RGD). (2) AI integration (wound segmentation, real-time auto-tuning of speed/pressure/photocuring and predicting all the steps of the bioprinting and healing processes). (3) Real-time sensors (on-device surface/depth OCT (Optical Coherence Tomography) mapping and optical monitoring to adapt deposition in situ; cf. in situ systems with integrated wound scanning) (e.g., [1]), hyperspectral scanning for analyzing tissue composition (e.g., oxygen level, infection presence, etc.) ([101,102]) and impedance monitoring of wound status ([103]). (4) Robotic autonomy (hand stabilization and semi-autonomous trajectories (operator draws paths, the robot adapts them); feasibility shown by an articulated collaborative (cobot) in situ bioprinter for skin wounds) (e.g., [81]). (5) Extreme environments (space-readiness with sterile sealed cartridges and microgravity-compatible workflows, as explored in ESA/DLR Bioprint FirstAid [20]), first-aid patches for warzones. (6) Standards & regulation (GMP-grade bioinks, sterility/validation, traceability and cybersecurity).

The next generation of handheld bioprinters will be shaped by the convergence of several technological pillars. Six interconnected domains—advanced smart bioinks, AI-based planning and control, integrated real-time sensors, automated robotic stabilization, space-ready design, and robust regulatory frameworks—collectively underpin future advances in personalization, autonomy, precision, and portability. Among these, smart bioinks and AI are discussed in the following subsections, as they already demonstrate direct translational impact on wound healing and treatment customization. Stimuli-responsive hydrogels with embedded growth factors or antimicrobial cues, for instance, can dynamically adapt to the wound environment to accelerate healing [79,80]. AI-driven wound analysis and closed-loop control further enable truly patient-specific patterning based on real-time imaging. Other directions, such as robotic hand stabilization [81], microgravity-qualified devices [20], photobiomodulation, and GMP-compliant validation protocols [14,15], illustrate how handheld systems may be extended to broader environments while ensuring safety and regulatory acceptance. Collectively, these innovations (Figure 3) point toward portable platforms capable of delivering highly personalized and autonomous regenerative interventions, even in the most demanding settings.

The integration of artificial intelligence (AI) with portable handheld bioprinters represents a novel approach to personalized medical treatment, particularly in emergency and remote settings. Such systems enable medical interventions tailored to individual patient characteristics, thereby improving both treatment efficacy and outcomes. AI analyzes patient-specific data, including genetic profiles and lifestyle factors, to customize therapy [82]. In oncology, for example, AI enhances precision by targeting tumors based on unique molecular features, enabling highly specific treatments [83]. Similarly, in personalized medicine, AI optimizes pharmacological regimens according to individual patient profiles, leading to improved therapeutic outcomes [84]. Although there is significant potential with AI and bioprinting, resolving issues of algorithmic bias and promoting regulatory steps is still required to ensure the proper application of these methods in clinical settings [83,84].

The development of “smart” healing materials, particularly for wound care, has been greatly accelerated by the use of stimuli-sensitive hydrogels and nanohydrogels. These materials respond to external stimuli (such as temperature, pH, and humidity) to create an optimal healing environment. According to various studies, stimuli-responsive hydrogels can sense and react to wound-specific cues, including pH, reactive oxygen species, glucose, and enzymes, as well as physical triggers like temperature and light. Such responsiveness facilitates targeted drug delivery and controlled release, both of which are critical for effective wound healing [79,80,85]. The incorporation of nanoparticles, including zinc oxide and iron oxide, further enhances hydrogel functionality by imparting antimicrobial properties that aid in infection control, tissue regeneration, and the modulation of inflammatory responses [86,87]. These nanoparticles also improve the mechanical strength and stability of the hydrogels, increasing their efficacy as wound dressings [86]. Additionally, intelligent materials—such as azobenzene-derived biomaterials and those exhibiting reversible optical, mechanical, and electrical switching—have been developed to respond to light, humidity, and temperature, offering self-healing and environment-responsive capabilities [88,89]. Their application in wound healing not only improves healing outcomes but also reduces the frequency of dressing changes, thereby enhancing patient comfort and lowering healthcare costs [86,87]. Overall, the creation of smart materials for active healing represents an exciting frontier in biomedical applications, with researchers leveraging their unique properties to design more efficient and responsive therapeutic products [90].

The incorporation of programmable release systems into such bioinks enables the controlled delivery of, e.g., mRNA and miRNA, thereby facilitating precise gene regulation during tissue regeneration. Hybrid bioinks composed of extracellular vesicles and liposomes have been shown to achieve sustained, targeted miRNA delivery within 3D-bioprinted tissues [91]. In tendon engineering, biomimetic GelMA-based bioinks combined with miRNA-16-5p and magnetic microfibers have been used to induce stem cell differentiation [92]. Photo-responsive polymeric nanoparticles have also been developed for controlled RNA release, offering accurate topical delivery and improved gene knockdown efficiency [93]. Moreover, angiogenic growth factors such as VEGF and PDGF-BB, which are essential for inducing angiogenesis and promoting tissue regeneration, can be incorporated into these systems. Aptamer-tethered systems, for instance, enable spatiotemporal control over growth factor release, thereby mimicking the natural dynamics of the extracellular matrix [94]. The inclusion of matrix proteins such as collagen, fibronectin, and gelatin further enhances cell interactivity by supporting adhesion, proliferation, and differentiation—processes that are critical for successful wound healing [95,96]. Self-healing capabilities have also become a priority, with hydrogels engineered to re-form covalent bonds upon exposure to water, ensuring the long-term integrity and functionality of the construct [97]. The future integration of biosensors into these materials promises real-time monitoring of the wound environment, enabling the delivery of personalized treatment regimens [98,99]. Moreover, 3D printing techniques further increase versatility by allowing hydrogel properties to be tailored to wound depth and type, thereby facilitating optimal healing [97]. In summary, the convergence of smart hydrogels with advanced biomaterials design and technology holds tremendous potential for personalized and efficient wound healing therapies [99,100].

## 7. Conclusions

The advancement of in situ bioprinting, particularly for skin regeneration, holds significant potential to transform clinical practice in reconstructive surgery and wound healing. Different handheld bioprinting modalities should therefore be regarded as complementary rather than universally interchangeable, with their suitability determined by the required mode of material deposition, bioink properties, wound geometry, and the level of spatial control needed for a given application. Overall, technological progress in handheld skin bioprinting continues to outpace clinical validation, with standardized performance criteria and robust human evidence remaining key requirements for translation. Key challenges for portable handheld bioprinters include the need for comprehensive mechanical testing and the establishment of standardized guidelines to ensure safe clinical application. At the same time, promising outcomes have been reported in preclinical studies, whereas therapeutic superiority of handheld skin bioprinting over standard care has not yet been established in controlled human studies. The emergence of portable handheld bioprinters represents a major step toward personalized, on-demand treatment, although obstacles related to material compatibility, regulatory approval, and market access remain. Emerging technologies, particularly the integration of artificial intelligence and advanced bioinks, may further support treatment personalization and the clinical development of these systems.

## Figures and Tables

**Table 3:** Current clinical trials involving handheld bioprinting techniques.

DOI/Trial ID	Title	Current Status	Country	Comments
NCT04890574	CellMist™ Autologous Cells to Treat Deep Second-Degree Burns (CELLMIST1)	Unknown Last update posted: 15 March 2022 Estimated study completion: 30 November 2022	USA	The goal of this multicenter pilot study is to evaluate the safety of the CellMist™ System for deep second-degree burns (≤30% TBSA) by spraying autologous epidermal/dermal cells onto debrided wounds using the SkinGun™ device. The device represents a handheld direct cell-spray approach rather than conventional layer-by-layer 3D bioprinting.
NCT04925323	A Dermo-Epidermal Autologous Skin Substitute For Further Therapeutic Use (BIOPSKIN)	Unknown Last update posted: 14 June 2021 Estimated study completion: October 2023	France	This study aims at generating GMP-compliant validation batches of ‘bio-printed dermo-epidermal substitutes” from 25 healthy volunteer patients’ unused surgical tissue removed during plastic surgeries. Ex vivo bioprinting study; not handheld or in situ.
ACTRN12625000088448; [22]	Safety and Feasibility Study of the Ligo In situ Bioprinting System (LIGO-SKIN-A001)	Initial first-in-human study reported ANZCTR last registry update: 28 January 2025	Australia	LIGO is a robotic direct in situ bioprinting system, rather than a handheld device. It deposits autologous skin cells and a two-component biomaterial directly into the wound, where the construct forms in situ.

Abbreviations: GMP—Good Manufacturing Practice.

**Table 4:** Comparison of the main features of handheld portable and conventional stationary bioprinters.

Parameter	Handheld Portable Bioprinter	Conventional Stationary Bioprinter
Resolution	100–500 µm Affected by hand motion and dynamic wound geometry	0.1–100 µm Precise control over layer thickness and x-y-z coordinates
Bioink Requirements	Requires rapid gelation, strong bioadhesion, shear-thinning behavior, and compatibility with on-device crosslinking	Can process a wider range of bioinks (low to high viscosity) Allows for complex multi-material constructs
Scalability	Optimized for localized wounds Coverage time and volume are constrained by physical operation and bioink cartridge size	Can fabricate large, pre-planned constructs in a controlled manner, making it suitable for large grafts and mass production
Portability	Battery-powered Designed for point-of-care, emergency, and field settings	Non-portable, requires a dedicated laboratory or cleanroom environment
Operational Complexity	Simple Designed for use by clinicians with minimal specialized training	Complex Requires skilled operators (bioprinting specialists) and complex software for design, slicing, and G-code generation
Cost	Prototypes ~$100–$500 Commercial research models ~$5000–$20,000 Operational costs are dependent on bioink reagents	Industrial systems $50,000–$500,000+ Requires high capital investment, specialized facilities, and trained personnel
Clinical Applicability	High for emergency/acute care Allows for immediate, patient-specific coverage of acute wounds, burns, and trauma	High for reconstructive care Ideal for scheduled surgeries (e.g., complex reconstruction, chronic ulcer treatment) where a custom graft can be pre-fabricated
Technology Maturity	Low–Moderate Mostly at prototype/preclinical stage (TRL 3–5) Few commercial research products exist	High Widely commercialized (TRL 6–9) Multiple systems available for research

**Table 5:** Key challenges in applying handheld bioprinters.

Challenge Type		Description
Material	Bioink limitations	do not sufficiently mimic the ultrastructure of the extracellular matrix
	Mechanical Properties	lack of the mechanical strength and integration
Technical	Printing precision and fidelity	low precision and accuracy because of print speed and nozzles size
	Cell viability assessment	challenging quantification of cell distribution in 3D
Methodological	Motion and volume restrictions	low complexity and small size

**Table 6:** Key market adoption barriers for applying handheld bioprinters.

Market Adoption Barrier	Description
Sterility and Bioprinter Hygiene	In surgery, handheld bioprinters must meet strict aseptic standards, yet complex hardware can harbor contaminants, making sterilization difficult [63]. Solutions include sterilizable materials, disposable nozzles, or sterile drapes [78]. Bioinks require sterile preparation and real-time contamination monitoring to prevent infection [63]. Regulatory approvals will demand validated sterility for both the device and printed tissues.
Operator Training and Human Factors	Adoption depends on ease of use and minimal training needs. Some designs, like Hakimi’s, allow surgeons to operate with little instruction [6]. Devices must be ergonomic, lightweight, and intuitive to avoid workflow disruption [63]. Where complexity exists, initial certification or training may still be needed to ensure consistent results.
Regulatory and Standardization	Handheld bioprinters involve devices and biologics, requiring clear frameworks for bioink formulation, printing, and tissue maturation. FDA guidance on additive manufacturing (2017) provides a starting point, but classification (device, biologic, or combination) affects approval paths. In the EU and elsewhere, compliance with medical device and tissue-engineering regulations is mandatory. Experts emphasize GMP-compliant production and robust quality standards as prerequisites for clinical translation [63].
Perception and Trust Concerns	The acceptance of portable bioprinting technology is also a matter of the attitudes of physicians and patients. Misperception or ignorance of the benefits and capabilities of such devices can create distrust and refusal in their use for clinic work. Establishing trust involves significant education and concrete evidence of safety and effectiveness, requiring time and extensive clinical proof [14].

## Data Availability

No new data were created or analyzed in this study. Data sharing is not applicable to this article.
